# Supplementary material for: Identification and functional analysis of non-coding regulatory small RNA FenSr3 in Bacillus amyloliquefaciens LPB-18
Source: PeerJ. 2023 May 15;11:e15236. doi: 10.7717/peerj.15236 (PMC10194069; doi:10.7717/peerj.15236)
Supplement: Supplemental Information 4 [file peerj-11-15236-s004.zip › KO/CK-vs-T1_map/map04068.html]

KEGG PATHWAY: FoxO signaling pathway - Reference pathway


|  |  |
| --- | --- |
| **FoxO signaling pathway - Reference pathway** |  |

[
Pathway menu
| Organism menu
| Pathway entry
| Show description
| User data mapping
]

The forkhead box O (FOXO) family of transcription factors regulates the expression of genes in cellular physiological events including apoptosis, cell-cycle control, glucose metabolism, oxidative stress resistance, and longevity. A central regulatory mechanism of FOXO proteins is phosphorylation by the serine-threonine kinase Akt/protein kinase B (Akt/PKB), downstream of phosphatidylinositol 3-kinase (PI3K), in response to insulin or several growth factors. Phosphorylation at three conserved residues results in the export of FOXO proteins from the nucleus to the cytoplasm, thereby decreasing expression of FOXO target genes. In contrast, the stress-activated c-Jun N-terminal kinase (JNK) and the energy sensing AMP-activated protein kinase (AMPK), upon oxidative and nutrient stress stimuli phosphorylate and activate FoxOs. Aside from PKB, JNK and AMPK, FOXOs are regulated by multiple players through several post-translational modifications, including phosphorylation, but also acetylation, methylation and ubiquitylation.

|  |  |  |
| --- | --- | --- |
| Reference pathway | 184% 150% 122% 100% 82% 67% 55% | 图片下载 |
